# The health quality of the diet and selected health indicators of Polish e-sports players

**DOI:** 10.3389/fnut.2026.1735909

**Published:** 2026-01-30

**Authors:** Paulina Mazur-Kurach, Maria Gacek, Aleksandra Pięta, Barbara Frączek

**Affiliations:** 1 Department of Sports Medicine and Human Nutrition, Institute of Biomedical Sciences, University of Physical Culture in Krakow, Krakow, Poland; 2 Department of Sports Dietetics, Gdansk University of Physical Education and Sport, Gdansk, Poland

**Keywords:** dietary health quality indicators, E-sports players, intestinal permeability markers, morphological and biochemical blood parameters, somatic characteristics

## Abstract

**Introduction:**

Diet is one of the important factors affecting health and physical performance. The aim of the study was to assess the relationship between the health quality of the diet and selected health indicators in a group of Polish e-sports players training at a professional and semi-professional level. The hypothesis was that a higher quality diet is associated with more favorable health indicators in e-sports players.

**Methodology:**

The study was conducted among 174 men aged 18–28 years, assessing anthropometric characteristics, morphological and biochemical blood biomarkers, and two markers of intestinal permeability in faeces. The Beliefs and Eating Habits Questionnaire KomPAN was used to assess diet quality. Statistical analysis of the relationships between diet quality indicators and health indicators was performed using Spearman’s rank correlation and principal component analysis (PCA) and multiple regression, assuming a test probability of *p* < 0.05.

**Results:**

The study group was dominated by e-sports with low levels of healthy diet indicators pHDI-10 (approx. 96%) and unhealthy diet indicators nHDI-14 (approx. 86%) and low overall diet quality DQI-24 (approx. 96%). Among the health indicators assessed, a low percentage of e-sports players had normal levels of uric acid and glucose in their blood (approx. 69 and 64%, respectively) and zonulin and lipopolysaccharides (LPS) in their faeces (37 and 28%, respectively). The Body Mass Index (BMI) was within the normal range for 60% of the group. Statistical analysis showed that the pHDI-10 index was significantly positively associated with vitamin D (*R* = 0.18) and HDL cholesterol (*R* = 0.19) and negatively with uric acid (*R* = −0.18) and blood glucose (*R* = −0.21) levels. The nHDI-14 index showed no significant associations with the analysed health indicators. However, the overall diet quality index DQI-24 was significantly negatively associated with uric acid levels (*R* = −0.18) and blood glucose (*R* = −0.23).

**Conclusion:**

A low level of dietary health quality, varied health indicators and significant correlations between dietary quality and certain health indicators were demonstrated, suggesting a positive impact of a high-quality diet on the health of Polish e-sports players.

## 1 Introduction

The development and professionalisation of e-sports has led to an increase in public awareness of the importance of health and a healthy lifestyle for sporting performance and the physical and psychological health of e-sports players (1, 2). Esports is a category of competitive video games which, despite obvious differences, can resemble traditional sports in many ways (3–5). Achieving success in e-sports requires, among other things, high cognitive abilities, reaction speed and mental resilience (6, 7). Esports players are also exposed to numerous biological and psychosocial health risks, including those related to lifestyle, such as poor nutrition, hypokinesia, psychological stress, etc. (1, 8–10). These lifestyle factors can adversely affect health, including somatic indicators, carbohydrate and lipid metabolism, and other physiological processes, such as gut microbiota composition and intestinal permeability, thereby increasing the risk of metabolic disorders and cardiovascular disease. (5, 9, 11). A sedentary lifestyle, prolonged sitting in front of a screen, and repetitive movements during many hours of gaming also increase the risk of neurological disorders and musculoskeletal disorders (9, 12–14). It is therefore important to promote a healthy lifestyle and health among e-gamers (15). One proposal is to integrate physical activity, psychological counselling, ergonomic optimisation, vision protection and health education, which may reduce health risks and promote the overall improvement of e-sports players’ health (9). Previous studies on the lifestyle of e-sports players have focused on, among other things, the level of physical activity and related physical fitness, as well as various health behaviors (16–20). These studies have shown a predominance of low and moderate levels of physical activity and an average level of physical fitness among e-sports players (20).

One of the important aspects of lifestyle that determines health, athletic ability and sporting success is a varied, balanced diet of high nutritional quality (21–23). A diet rich in products with a high content of bioactive substances with nutraceutical properties, including polyphenols, antioxidant vitamins, dietary fibre, probiotics, Polyunsaturated Fatty Acids omega-3 (PUFAs) and other ingredients with proven health-promoting properties, including those related to the regulation of metabolic processes, pro-oxidative-antioxidative balance, immune resistance, lipid and carbohydrate metabolism, etc. (22–24). A high-quality diet is associated with regular consumption of fruit and vegetables, legumes, whole grain products, fermented dairy products and sea fish, which contain ingredients important for maintaining antioxidant status and acid–base balance, regulating intestinal peristalsis and gut microbiota, and optimising blood lipid profile. On the other hand, preferring foods with low nutrient density, including those rich in sugars, trans fatty acids, cholesterol and trans isomers, reduces the nutritional value and health quality of the diet. An important aspect of a healthy diet is also a balanced energy supply that promotes a sustainable energy balance and thus a healthy body weight (21–23). A high-quality healthy diet, based on the principles of the Mediterranean diet, can reduce the risk of health problems, including metabolic and cardiovascular disorders, to which e-sports players are exposed (25, 43). The health quality of a diet can be assessed using various indicators, including the Healthy Eating Index (HEI), the Australian Diet Quality Index (Aussie-DQI) (26, 27), as well as the health-promoting diet index (pHDI-10), unhealthy diet index (nHDI-14) and overall diet quality index (DQI-24) (28). The healthy diet index refers to the frequency of consumption of products with a potentially beneficial effect on health, the unhealthy diet index refers to the consumption of products with a potentially negative effect on health, and the overall diet quality index is a compilation of both of these indices (28).

Despite the key role of diet in health and sporting success, including in e-sports, studies from various research centres have revealed nutritional irregularities, both quantitative and qualitative, in athletes training in various individual and team sports (29–31). Previous work in this area among e-sports players has primarily focused on the nutritional behaviors and patterns of Polish, German, Brazilian, Portuguese and Turkish e-gamers (16, 32–35) and the relationship between diet and the state of the gut microbiota (36). Earlier Polish studies have shown low and moderate levels of rational eating behaviors (34) and the predominance of unhealthy eating patterns among e-gamers (35). Turkish studies have also shown the prevalence of dietary abnormalities, including high consumption of alcohol, energy drinks and processed foods, which may increase the risk of obesity and metabolic disorders in e-sports players (16). The importance of diet for health and competitive ability, including cognitive ability (37), in light of previous studies suggesting dietary irregularities in people training in e-sports, was one of the reasons for undertaking the presented research.

In this context, given the importance of nutrition for health and, indirectly, for sporting performance, as well as the well-established relationship between diet and health, this study aimed to assess selected nutritional determinants of health-related indicators in a group of Polish professional and semi-professional e-sports players. The following research questions were developed:

1. What is the health quality of the diet of Polish e-sports players?
2. What are the BMI and other health indicators (morphological and biochemical blood tests, intestinal permeability) of Polish e-sports players?
3. What are the relationships between diet quality indicators and BMI and other health indicators in Polish e-sports players?

The hypothesis was that a higher quality diet is associated with more favorable health indicators in e-sports players.

## 2 Materials and methods

### 2.1 Participants

The study was conducted between 2022 and 2024 in a group of Polish e-sports players. The study was conducted on a group of 174 male aged 18–28 (20 ± 3) years, recruited from the central region of Poland, including those training at a professional level (*n* = 44) and semi-professional level (*n* = 130). *A priori* sample size estimation was performed using G*Power software. Assuming a medium effect size (Cohen’s *d* = 0.5), a two-tailed significance level of *α* = 0.05, and a desired statistical power of 0.80, the minimum required sample size was estimated at approximately 64 participants, so the study sample meets the required size criterion. Unlike semi-professional players, professional players had professional experience, defined as receiving financial rewards and undergoing specialised training. Professionals spent more time playing computer games than semi-professionals (8 ± 2 h day^−1^ vs. 6 ± 1 h day^−1^). The players surveyed had at least 5 years of training experience (6 ± 2) and participated in e-sports tournaments, including Counter-Strike: Global Offensive (CS: GO), League of Legends (LoL) and StarCraft. The group consisted of university students (33%), secondary school students (23%) and working professionals (44%).

The following criteria were adopted for selecting participants for the study: practising e-sports for at least 5 years and undertaking regular training at least 5 times a week in 5–6 h training sessions. The exclusion criteria included: lack of informed written consent to participate in the study, age below 18 and above 40, and female gender. The study was conducted in accordance with the principles of the 1964 Helsinki Declaration, after obtaining the informed written consent of the participants, and the study protocol was approved by the Bioethics Committee for Scientific Research in Gdańsk (KB-63/22).

### 2.2 Research tools

#### 2.2.1 Assessment of the health quality of the diet (healthy diet index pHDI-10, unhealthy diet index nHDI-14 and overall diet quality index DQI-24)

The standardized Beliefs and Eating Habits Questionnaire created by the Behavioral Conditions of Nutrition Team, Committee of Human Nutrition Science, Polish Academy of Science (28, 38) was used to assess diet quality. The frequency of consumption of products was assessed on a 6-point scale with ranks ranging from (6)_several times a day, to (1)_never. These ranks were then converted into actual numbers representing the daily frequency of consumption, according to the following scheme: (2)_several times a day, (1)_once a day, (0.5)_several times a week, (0.14)_once a week, (0.06)_several times a month, (0)_never (28, 38). By summing up the values determining the daily frequency of consumption of products, the Pro-Healthy Diet Index (pHDI-10) and the Non-Healthy Diet Index (nHDI-14) were calculated. The pHDI-10 index is determined by the frequency of consumption of 10 product groups: vegetables, fruit, legumes, wholemeal bread, other whole meal cereal products, milk, fermented milk drinks, cottage cheese, white meat and fish. The nHDI-14 index is determined by the frequency of consumption of 14 product groups: white bread, other refined cereal products, fried foods, butter, lard, yellow and processed cheeses, meat products, red meat, canned meat, fast food, sweets, sweetened beverages, energy drinks and alcoholic beverages (28, 38). In interpreting the results, it is assumed that the higher the index value, the higher the intensity of nutritional characteristics that are beneficial or detrimental to health. To simplify the interpretation, the values of both indices expressed as multiples/day were converted to a 0–100 point scale (with 0–33 interpreted as low, 34–66 as moderate and 67–100 as high), in accordance with the methodology described (28, 38). The overall diet quality index (DQI-24) was also calculated by summing up all components of a healthy diet (pHDI-10) (with a positive sign) and an unhealthy diet (nHDI-14) (with a negative sign), according to the formula: DQI-24 = (100/20) x sum of the daily frequency of consumption of 10 food groups with a potentially positive effect on health + (−100/28) x sum of the daily frequency of consumption of 14 food groups with a potentially adverse effect on health. The DQI is interpreted as follows: a score between −100 and −26 points indicates a high intensity of unhealthy diet characteristics, a score between −25 and 25 points indicates a low intensity of unhealthy and healthy diet characteristics, and a score between 26 and 100 points indicates a high intensity of healthy diet characteristics (28).

#### 2.2.2 Measurement of somatic characteristics

Height was measured to the nearest 0.1 cm using a Holtain (UK) anthropometer, and body weight was measured using a Tanita TBF300 (Japan) electronic scale. Body mass index (BMI) was calculated using the formula: body weight/height (kg/m^2^). BMI was interpreted according to the World Health Organization criteria, with values of 18.50–24.99 kg/m^2^ classified as normal weight, 25.00–29.99 kg/m^2^ as overweight, and ≥30.00 kg/m^2^ as obesity. Somatic measurements were performed by the authors of the study in the morning hours, in a fasting state, after bladder emptying. Participants were measured barefoot and wearing light clothing, according to standard anthropometric procedures.

#### 2.2.3 Measurement of intestinal permeability markers

Lipopolysaccharides (LPS) and zonulin were used as markers of intestinal permeability. Zonulin reflects the regulation of intestinal tight junctions and intestinal barrier integrity, whereas LPS, a component of the outer membrane of Gram-negative bacteria, serves as an indicator of increased intestinal permeability and translocation of bacterial endotoxins (36). Intestinal permeability markers (LPS and zonulin) were assessed in stool samples collected by e-sports players from three locations in order to standardize the samples. The material was placed in sterile containers, which were stored in a cool place until delivery to the laboratory. The tests were performed using the ELISA technique at Alab Laboratories (Warsaw, Poland). Stool sample collection was performed according to a standardized protocol. Participants were provided with sterile, single-use containers and detailed instructions for sample collection. Stool samples were self-collected by the participants under home conditions, following hygienic procedures and avoiding contamination with urine or water. Samples were collected from different sites of the same stool specimen to obtain a representative sample. After collection, the samples were immediately cooled, transported to the laboratory, and stored at −80 °C until analysis. Concentrations of zonulin and lipopolysaccharide (LPS) were determined using enzyme-linked immunosorbent assay (ELISA) methods, in accordance with the manufacturers’ instructions.

#### 2.2.4 Measurement of morphological and biochemical blood parameters

Venous blood samples were collected from the antecubital vein after an overnight fast. Hematological and biochemical biomarkers of peripheral blood were assessed in samples taken from the antecubital vein. The following hematological and biochemical parameters were assessed: hematocrit (HCT); hemoglobin (HGB); erythrocytes; platelets; leukocytes; glucose; total cholesterol (TC); HDL and LDL lipoproteins; triglycerides (TG); creatinine; uric acid (UA); total protein; albumin; liver enzymes, i.e., alanine aminotransferase (ALT), aspartate aminotransferase (AST), acid phosphatase, gamma-glutamyl transpeptidase (GGTP); total bilirubin; sodium; potassium; chloride; calcium; magnesium; phosphorus; iron; ferritin; vitamin D and vitamin B12. Blood tests were performed using flow cytometry (complete blood count), the erythrocyte sedimentation rate kinetic-photometric method (ESR), indirect potentiometry (Na, K, chloride), spectrophotometry (total protein, calcium, phosphorus, magnesium, TC, HDL, TG, UA, iron, AST, ALT, creatinine, GGTP, glucose) and direct chemiluminescence (vitamin B12, vitamin D, ferritin). Laboratory tests were performed using the following devices: AlinityHQ (Abbott, Singapore, Singapore), Roller 20 (Alifax, Polverara, Italy), Alinity C (Abbott, Singapore, Singapore), Alinity I (Abbott, Singapore, Singapore) and Atellica 1,500 (Siemens, Munich, Germany), as an external service provided by AlabLaboratories (Warsaw, Poland).

### 2.3 Statistical analyses

Statistical analyses were performed using Statistica 13 and Excel. Basic descriptive statistics were compiled (M—mean; SD—standard deviation; Me—median; Min—minimum; Max—maximum; Q25—lower quartile; Q75—upper quartile; values are presented as percentages where appropriate, depending on the type of variable). The normality of the data distribution was checked using the Shapiro–Wilk test. Statistical analysis of the relationships between diet quality indicators and health indicators was performed using Spearman’s non-parametric rank correlation. The “*R*” values denote Spearman’s correlation coefficients, used to assess the strength and direction of associations between dietary indices and biochemical parameters, with positive values indicating direct relationships, negative values indicating inverse relationships, and higher absolute values reflecting stronger correlations The strength of the correlation increases as the absolute value of the correlation coefficient (*R*) approaches 1, with values close to 0 indicating weak associations and values closer to ±1 indicating strong associations (39). In order to examine the complex relationships between variables, a multidimensional statistical analysis was also performed. In this regard, multiple regression analysis and principal component analysis (PCA) were performed, with the Kaiser method and a scree plot used to determine the number of principal factors. Factor rotation was performed using the normalized Varimax method. Statistical significance was set at *α* = 0.05 and the test probability at *p* < 0.05.

## 3 Results

### 3.1 Health quality of the diet of polish e-sports players

Among products with potentially beneficial health effects, the highest percentage of e-sports players surveyed consumed vegetables (36.43%), fruit (35.00%) and milk (35.72%) at least once a day. Among products with a potentially adverse effect on health, the highest percentage of e-gamers surveyed consumed white bread (65.00%), butter (39.29%) and meat products (28.57%) at least once a day (Table 1).

**Table 1:** Frequency of consumption of products with a potentially beneficial (pHDI-10) and potentially adverse (nHDI-14) effect on health among Polish e-sports players (percentage of respondents).

Food products		Frequency of consumption					
		1	2	3	4	5	6
Potential health benefits (pHDI-10)	Fruit	4.29	9.29	18.57	32.86	20.00	15.00
	Vegetables	4.29	5.71	16.43	36.43	22.86	13.57
	Wholemeal bread	37.14	20.00	15.71	19.29	2.86	5.00
	Coarse groats, oat flakes, wholemeal pasta	30.71	38.57	18.57	10.00	0.71	0.71
	Legumes	39.29	29.29	16.43	11.43	2.14	0.00
	Milk	9.29	8.57	15.71	29.29	22.86	12.86
	Fermented milk products	19.29	20.00	22.86	26.43	7.14	3.57
	Cottage cheese	29.29	32.14	20.71	10.71	5.00	1.43
	Poultry dishes	2.86	7.86	20.00	52.86	14.29	0.71
	Fish dishes	26.43	29.29	16.43	11.43	2.14	0.00
Potentially adverse health effects (nHDI-14)	White bread	2.85	3.57	11.43	16.43	26.43	38.57
	White rice, small groats	2.86	10.71	25.71	44.29	10.00	5.00
	Yellow cheese, blue cheese, processed cheese	9.29	11.43	25.71	26.43	19.29	5.71
	Cold cuts, sausages, frankfurters	7.89	10.71	15.00	35.71	15.00	13.57
	Red meat dishes	18.57	35.00	22.14	17.86	3.57	0.71
	Canned meat	25.71	29.29	15.00	24.29	2.86	2.14
	Fried dishes	2.86	4.29	13.57	57.14	18.57	2.87
	Butter	9.29	15.00	15.00	19.29	27.86	11.43
	Lard	95.00	4.29	0.00	0.00	0.00	0.71
	Fast food	6.43	35.00	32.86	21.43	2.86	0.00
	Sweets and confectionery	7.14	14.29	22.14	35.00	13.57	5.71
	Sweet carbonated drinks and non-carbonated drinks	13.57	20.71	22.14	24.29	8.57	8.57
	Energy drinks	27.86	27.86	25.00	9.29	5.0	2.86
	Alcoholic beverages	32.86	37.86	19.29	6.43	1.43	0.71

(1)__Never (2)__1–3 times a month (3)__Once a week (4)__Several times a week (5)__Once a day (6)__Several times a day.

Table 2 presents the values of the health diet quality indices of the surveyed e-sports players. The median value of the healthy diet index (pHDI-10) was 14.50 points, the unhealthy diet index (nHDI-14) was 18.89 points, and the overall diet quality index (DQI-24) was −4.34 points.

**Table 2:** Health quality index values for Polish e-sports players (descriptive statistics).

Diet quality indices	M ± SD	Me (Q25; Q75)	Min–Max
pHDI-10 (multiplicity/day)	3.09 ± 1.69	2.90 (1.75; 3.98)	0.00–10.20
nHDI-14 (multiplicity/day)	5.80 ± 2.83	5.43 (3.27; 7.51)	0.50–14.56
pHDI-10 (points)	15.48 ± 8.43	14.50 (8.75; 19.90)	0.00–51.00
nHDI-14 (points)	20.72 ± 10.12	18.89 (11.69; 26.82)	1.79–52.00
DQI-24 (points)	−5.21 ± 10.41	−4.34 (−9.92; 1.17)	−32.86–18.83

M_arithmetic mean, SD_standard deviation, Me_median, Q25_lower quartile, Q75_upper quartile.

The study group was dominated by e-sports players with low pHDI-10 healthy diet scores (96.43%) and low nHDI-14 unhealthy diet scores (86.43%), as well as low DQI-24 overall diet quality scores (95.71%) (Table 3).

**Table 3:** Level of healthy diet quality indices among polish e-sports players (numbers and percentages).

Indicators	Level	*N*	%
pHDI-10	Low (0–33 points)	135	96.43
	Moderate (34–66 points)	5	3.57
	High (67–100 points)	0	0.00
nHDI-14	Low (0–33 points)	121	86.43
	Moderate (34–66 points)	19	13.57
	High (67–100 points)	0	0.00
DQI-24	High intensity of unhealthy traits (−100 to −26 points)	6	4.29
	Low intensity of unhealthy and healthy characteristics (−25 to 25 points)	134	95.71
	High intensity of health-promoting characteristics (26–100 points)	0	0.00

### 3.2 Selected health indicators of polish e-sports players

Table 4 presents descriptive statistics for the analyzed health indicators in terms of blood morphological and biochemical parameters, intestinal permeability markers and BMI against reference values. The results showed that the majority of the e-sports players surveyed (over 90%) had normal values for hematological indicators (erythrocyte, hemoglobin, platelet and hematocrit levels). Over 90% of the e-sports players also had normal levels of chloride, magnesium, iron and ferritin, as well as normal activity of AST, GGTP, bilirubin and creatinine in their blood. Less than 70% of the men tested had normal levels of UA and fasting glucose. The lowest percentage of e-sports players had normal levels of intestinal permeability indicators, i.e., zonulin and LPS in faeces (37.00 and 28.00%, respectively). Normal body mass index (BMI) values were observed in 60.00% of the male participants. Among the remaining subjects (40.00%), overweight was the most prevalent condition (22.00%), whereas underweight (11.00%) and obesity (8.00%) were less frequently identified.

**Table 4:** Values of the analysed health indicators of Polish e-sports players (descriptive statistics).

Analysed parameters	M ± SD	Me (Q25; Q75)	Min–Max	Reference values^a^	Standard (% of group)
Leukocytes (×10^9^/L)	6.97 ± 1.78	6.60 (5.86; 7.91)	3.26–14.06	4.0–10.0	89.14
Erythrocytes (×10^12^/L)	5.17 ± 0.31	5.18 (4.91; 5.37)	4.47–5.89	4.5–5.9	90.86
Haemoglobin (g/dL)	15.25 ± 0.87	15.30 (14.70; 15.90)	11.60–17.60	13.5–17.5	93.71
Haematocrit (%)	44.19 ± 2.31	44.20 (42.70; 45.90)	35.90–50.30	40.0–50.0	92.57
Platelets (×10^9^/L)	242.27 ± 51.09	239.0 (209.00; 271.00)	121.00–402.00	150–400	92.57
Albumin (g/dL)	4.96 ± 0.24	5.00 (4.80; 5.10)	4.40–5.40	3.5–5.0	66.86
Total protein (g/dL)	7.26 ± 0.94	7.10 (6.90; 7.38)	6.40–12.51	6.0–8.0	89.71
Calcium (mmol/L)	9.58 ± 0.38	9.59 (9.35; 9.82)	8.69–10.66	2.15–2.55	82.86
Chlorides (mmol/L)	101.68 ± 1.83	101.65 (100.33; 102.90)	96.40–107.90	98–107	92.00
Phosphorus inorganic (mmol/L)	4.15 ± 0.63	4.11 (3.77; 4.49)	2.33–6.09	0.81–1.45	88.50
Magnesium (mmol/L)	2.15 ± 0.12	2.15 (2.06; 2.21)	1.90–2.60	0.65–1.05	94.29
Iron (μmol/L)	84.15 ± 34.67	78.00 (59.00; 102.00)	11.00–208.00	1–31	92.00
Ferritin (μg/L)	109.23 ± 60.09	97.40 (66.90; 139.80)	9.40–321.80	30–400	93.14
Vitamin B12 (pg/mL)	380.99 ± 122.60	369.80 (291.20; 463.50)	133.10–774.10	200–900	87.43
Vitamin D (ng/mL)	18.11 ± 8.46	16.60 (11.20; 24.30)	6.00–42.60	30–50	73.00
Sodium (mmol/L)	140.96 ± 1.86	141.00 (140.00; 142.00)	135.00–147.00	135–145	90.85
Potassium (mmol/L)	4.25 ± 0.34	4.22 (4.02; 4.43)	3.38–5.41	3.5–5.1	89.71
ALT (U/L)	24.75 ± 21.61	18.00 (12.00; 27.00)	5.00–133.00	7–56	88.00
AST (U/L)	22.24 ± 13.31	19.00 (16.00; 24.00)	10.00–138.00	10–40	95.43
GGTP (U/L)	20.35 ± 14.92	15.00 (12.00; 21.00)	6.00–107.00	10–71	95.43
Total bilirubin (mg/dL)	10.62 ± 5.87	9.58 (6.58; 12.71)	2.34–43.40	0.3–1.2	96.00
Uric acid (μmol/L)	378.74 ± 74.66	371.20 (328.00; 418.50)	217.20–633.00	210–420	69.14
Creatinine (μmol/L)	82.50 ± 10.87	82.80 (76.25; 88.55)	48.00–113.30	62–115	90.27
Fasting glucose (mmol/L)	5.32 ± 0.44	5.34 (5.09; 5.59)	2.90–6.52	3.9–5.5	64.00
Total cholesterol (mmol/L)	148.32 ± 29.05	145.00 (129.00; 165.00)	77.00–227.00	<5.0	78.29
HDL (mmol/L)	47.55 ± 9.88	47.40 (40.90; 54.00)	28.50–75.00	>1.0	75.43
LDL (mmol/L)	81.63 ± 27.05	78.00 (65.00; 96.00)	11.00–163.00	<3.0	89.14
Triglycerides (mmol/L)	92.32 ± 45.47	81.00 (60.00; 115.00)	24.00–285.00	<1.7	87.43
LPS in faeces (EU/ml)	0.69 ± 0.21	0.68 (0.55; 0.79)	0.25–1.37	>0.63	28.00
Zonulin in faeces (ng/ml)	68.15 ± 25.73	66.30 (51.49; 79.72)	12.50–156.39	<65	37.00
BMI (kg/m^2^)	23.45 ± 4.43	23.07 (20.20; 26.11)	15.23–40.11	18.5–24.9	60.00

a Ostrowska et al. (71).

M_arithmetic mean, SD_standard deviation, Me_median, Q25_lower quartile, Q75_upper quartile.

### 3.3 Relationships between the health quality of the diet and health indicators of polish e-sports players

Statistical analysis showed that the healthy diet index (pHDI-10) was significantly positively associated with vitamin D concentration (*R* = 0.18) and HDL cholesterol level (*R* = 0.19) and negatively with UA levels (*R* = −0.18) and blood glucose levels (*R* = −0.21). The unhealthy diet index (nHDI-14) showed no significant correlations with the analyzed health indicators. However, the overall diet quality index (DQI-24) was significantly negatively associated with UA levels (*R* = −0.18) and blood glucose levels (*R* = −0.23) (Table 5).

**Table 5:** Relationships between dietary quality indicators and health indicators in Polish e-sports players (Spearman’s *R* correlations).

Diet quality indicators and health indicators	pHDI-10		nHDI-14		DQI-24	
	*R*	*p*	*R*	*p*	*R*	*p*
Leukocytes	−0.09	0.31	0.01	0.94	−0.08	0.38
Erythrocytes	−0.13	0.16	−0.07	0.46	−0.04	0.67
Haemoglobin	−0.14	0.13	−0.08	0.39	−0.04	0.69
Haematocrit	−0.13	0.17	−0.05	0.56	−0.05	0.58
Platelets	0.02	0.87	0.05	0.58	−0.03	0.87
Albumin	−0.01	0.96	0.09	0.60	−0.09	0.58
Total protein	−0.02	0.13	0.07	0.46	−0.08	0.38
Total calcium	0.07	0.45	0.05	0.56	0.01	0.95
Chlorides	0.06	0.53	−0.10	0.31	0.13	0.15
Inorganic phosphorus	0.04	0.64	0.08	0.38	−0.04	0.67
Magnesium	0.03	0.75	0.11	0.24	−0.08	0.41
Iron	0.03	0.74	−0.06	0.52	0.08	0.40
Ferritin	−0.10	0.25	0.00	0.96	−0.08	0.39
Vitamin B12	−0.02	0.85	0.00	0.99	−0.01	0.89
Vitamin D	0.18*	0.05	0.02	0.80	0.12	0.19
Sodium	−0.08	0.41	−0.08	0.37	0.02	0.85
Potassium	0.10	0.27	0.01	0.93	0.07	0.44
ALT	0.01	0.94	−0.03	0.73	0.03	0.71
AST	0.06	0.48	0.00	0.97	0.05	0.61
GGTP	−0.14	0.11	−0.21	0.12	0.08	0.39
Total bilirubin	0.02	0.89	0.01	0.89	0.00	0.99
Uric acid	−0.18*	0.05	0.06	0.48	−0.18*	0.04
Creatinine	−0.04	0.68	−0.07	0.44	0.03	0.70
Fasting glucose	−0.21*	0.02	0.08	0.41	−0.23**	0.01
Total cholesterol	−0.03	0.90	−0.04	0.66	0.01	0.72
HDL cholesterol	0.19*	0.04	−0.13	0.12	0.04	0.68
LDL cholesterol	−0.09	0.32	0.13	0.15	0.05	0.32
Triglycerides	−0.12	0.20	−0.06	0.51	−0.04	0.69
LPS in faeces	0.11	0.25	0.08	0.38	0.01	0.92
Zonulin in faeces	−0.09	0.57	−0.09	0.56	0.03	0.85
BMI	0.06	0.65	−0.13	0.15	0.06	0.65

^*^p ≤ 0.05; ^**^p ≤ 0.01.

The multidimensional analysis did not reveal any statistically significant correlations between the health quality of the diet, assessed on the basis of the pHDI-10, nHDI-14, and DQI-24 indices, and the health indicators of Polish e-sports players (*p* > 0.05).

## 4 Discussion

The study showed that the diet was not very healthy, with different levels of the health indicators analyzed and links between diet quality and some health biomarkers of Polish e-sports players. The results obtained allowed for a partially positive verification of the research hypothesis that higher dietary health quality is positively associated with health, although significant associations were found for only a few of the analyzed health indicators, and the strength of these associations was low. The weak correlations between the healthy diet index (pHDI-10) and the overall diet quality index (DQI-24) and certain health biomarkers of Polish e-sports players correspond to the lack of significant correlations between these variables in the multivariate analysis models, which suggests that the results should be interpreted with caution.

### 4.1 Health quality of the diet of e-sports players

The Polish e-sports players surveyed were found to have a low level of dietary health quality, with low scores recorded for both the healthy diet index (pHDI-10) and the unhealthy diet index (nHDI-14) as well as the overall dietary quality index (DQI-24). The categorization of the overall diet quality index confirmed that the vast majority of the e-gamers surveyed showed low levels of both healthy and unhealthy dietary characteristics. These results should be interpreted as a low positive and low negative impact of the diet on health. Referring to the detailed results obtained in terms of the frequency of consumption of products defining the analyzed diet quality indicators, it can be concluded that the described low level of health quality of the e-sports players’ diet was associated with insufficient consumption of products recommended in the diet, including fruit and vegetables, whole grain products, legumes, fermented dairy products, cottage cheese and sea fish, and at the same time with high consumption of products not recommended in the diet, including in particular processed meat products, fried foods, sweets and confectionery. Meanwhile, a diet rich in nutraceuticals, including antioxidant vitamins, polyphenols, dietary fiber, probiotics and omega-3 PUFAs, is extremely important for health as it helps to reduce oxidative stress, regulate intestinal peristalsis (which is weakened by a sedentary lifestyle), limit the direct and long-term effects of intestinal dysbiosis and optimize the blood lipid profile (24, 40–45). At the same time, a high intake of saturated fatty acids, simple sugars and trans isomers may increase health risks, including those associated with the development of obesity, type 2 diabetes and cardiovascular disease (46). Nutritional errors can also negatively affect the cognitive abilities of e-sports players through an excess of processed foods rich in saturated fatty acids and simple sugars and a deficiency of certain vitamins (B and D) and minerals (iodine, zinc, selenium, iron, magnesium, phosphorus, potassium) and bioactive substances (antioxidants and probiotics) (37, 47, 48).

The low level of dietary health quality among Polish e-sports players demonstrated in the studies discussed corresponds to the results obtained in this regard in other population groups, including Polish athletes training among team sports (49) and Polish and Spanish physical education students (50). The poor health quality of the diet of the e-sports players surveyed is also in line with the trends described in among various groups of e-sports players. Earlier studies among Polish e-sports players have shown abnormal eating habits, particularly in terms of low consumption of foods with high nutritional value, including vegetables, whole grains, legumes and sea fish, which translated into low and average levels of compliance with nutritional recommendations (34). Previous studies have also shown a predominance of unhealthy eating patterns among Polish e-sports players, including patterns described as “fast food,” “highly processed foods, meat and sweets,” “sweets” and “fats—dairy products” and “fats,” with only one healthy pattern (“vegetables-fruit”) (35). Turkish studies have also confirmed dietary irregularities among e-sports players, including high consumption of alcoholic and energy drinks and processed foods, which may increase the risk of obesity and metabolic disorders in this population group (16). German video gamers and e-sports players have also been reported to have dietary irregularities, including high consumption of energy drinks and fast food products and low consumption of vegetables and fruit (33). Low consumption of vegetables and fruit has also been described among German, Portuguese and Brazilian e-sports players (32, 51). American studies among electronic gamers have also shown nutritional abnormalities, both quantitative (deficiencies in B vitamins and certain minerals – magnesium, potassium, zinc and selenium) and qualitative (low consumption of vegetables, fruit and dairy products), indicating a need for dietary rationalization and nutrition education among e-sports players (47). Results indicating qualitative and quantitative nutritional deficiencies have also been reported in traditional athletes, including Polish team sports athletes (30, 31). A new literature review has also shown that the quality of athletes’ diets is suboptimal and needs to be improved in terms of the consumption of whole grains, fruit and dairy products in order to reduce the risk of chronic diseases and support recovery processes (52).

### 4.2 Selected health indicators for e-sports players

The studies discussed showed varying levels of the analyzed health indicators in terms of morphological and biochemical blood parameters, intestinal permeability markers and BMI in Polish e-sports players. It was shown that the vast majority (over 90%) of the e-sports players studied had normal hematological indicators and parameters related to liver (AST, GGTP, bilirubin) and kidney function (creatinine) and normal levels of certain elements (magnesium and iron) and ferritin. The majority (over 80%) also had normal levels of total protein, calcium, phosphorus, electrolytes (sodium and potassium), vitamin B12, LDL and triglycerides. At the same time, however, over 30% of e-sports players had elevated blood glucose and UA levels, 27% had low vitamin D 25(OH) levels, and over 20% had abnormal blood lipid profiles (excess total cholesterol and low HDL levels). At the same time, 40% of the male subjects had an above-normal BMI. The study group also had a particularly high percentage of players with abnormal intestinal barrier permeability parameters (63.00% for zonulin and 72.00% for LPS).

The designated level of the analyzed health indicators, including those related to nutritional status, points to fundamental problems and potential health risks in this population group, including those related to lifestyle due to the specific nature of the e-sport discipline practiced. In the context of the results obtained, the presence of hyperglycemia, hypercholesterolemia, hyperuricemia and excessive body weight can be indicated in the study group, increasing the risk of developing chronic diseases, including type 2 diabetes and cardiovascular diseases. The described metabolic disturbances may be associated not only with poor diet quality but also with other lifestyle factors, including low levels of physical activity, as demonstrated in previous studies (20). The risk of developing chronic diseases in the e-sports players studied may also be exacerbated by increased intestinal permeability, indicating potential disturbances in the intestinal microbiota and reduced intestinal barrier integrity. Dysbiosis and increased intestinal permeability can lead to the development of chronic diseases, including cardiometabolic diseases, inflammation, reduced immune function, and cognitive impairment in e-sports players (44, 53, 54, 37, 58). In turn, vitamin D (marked as calcidiol) deficiencies can disrupt calcium and phosphate metabolism and increase the risk of developing osteoporosis (55). To summarize the scale of disorders in the analysed health indicators among Polish e-sports players, it should be noted that intestinal permeability disorders were the most prevalent. A tendency towards increased levels of intestinal permeability markers has also been described among players from various disciplines, including Polish athletes (56) and elite Italian footballers (57). It can therefore be concluded that, despite obvious differences in sporting characteristics, elevated levels of intestinal permeability markers occur in both e-sports players and traditional sports athletes. Another health problem in the group of e-players studied was the prevalence of excess body weight, which may also be related to a sedentary lifestyle. Tendencies towards low physical activity and increased body fat composition have been described in American e-sports players (19). Low levels of physical activity, conducive to weight gain, have also been described in Polish e-sports players (20). The risk of excessive body weight and metabolic disorders in e-gamers has also been indicated in Turkish studies (16). A current review of the literature in this area has confirmed that overweight and obese individuals have elevated levels of zonulin, indicating increased intestinal permeability (58). It should also be noted that the gut microbiota, by influencing energy metabolism, depending on the state of eubiosis or dysbiosis, can have a positive or negative effect on body weight and composition (59). Previous studies have shown a positive correlation between BMI and intestinal permeability markers (36), which is consistent with the trends described above. In light of our own research and that of other authors, it therefore seems important to promote a healthy, active lifestyle that reduces the risk of carbohydrate and lipid metabolism disorders, excessive body weight and its complications, as well as other proven health risks in this population group (5, 9).

There are significantly more studies in the literature on health indicators, including biochemical, hematological and metabolic parameters, in traditional athletes than in e-sports players. These studies indicate variability in basic biochemical and hematological values in e-sports players compared to the general population (most notably in the case of creatine kinase CK), high values of other blood indicators (AST, ALT, Lactate Dehydrogenase LDH, potassium and total bilirubin) (60). It appears that, for e-sports players, the general population may represent a more appropriate reference group than elite athletes. Health studies conducted in a large cohort of Olympic athletes from 2012 to 2024 also revealed numerous biochemical abnormalities and metabolic disorders, including dyslipidemia (20%), hypercortisolemia (15%), iron deficiency (approx. 10%) and glucose intolerance (approx. 8%), with dyslipidemia and glucose intolerance being more common in males than females. It can therefore be concluded that certain health disorders affect both traditional athletes and e-sports players. In light of the results of our own research and data from the literature, it seems that blood tests in traditional athletes and e-sports players should be an important area of health prevention (61). The literature also highlights the role of biomarkers (including inflammation and oxidative stress) in monitoring chronic fatigue in professional athletes (62). It seems that similar studies, extended to include mental health assessments, could also be applied to e-sports players.

### 4.3 Relationships between the health quality of diet and health indicators in e-sports players

An analysis of the relationships between the health quality of the diet and the health of e-sports players showed that as the healthiness of the diet (pHDI-10) increased, the levels of vitamin D 25(OH) and HDL lipoprotein fractions increased, while the levels of UA and glucose in the blood decreased. The unhealthy diet index (nHDI-14) showed no significant correlation with the health indicators analyzed. At the same time, as the overall diet quality index (DQI-24) increased, blood UA and glucose levels decreased. However, it should be noted that the significant correlations found between diet quality and certain health parameters were weak (R in the range of 0.18–0.23), which requires cautious interpretation and conclusion, especially since multidimensional analysis did not reveal a significant predictive role of the assessed diet quality for the health of e-sports players. The results obtained allow for a partially positive verification of the research hypothesis, with the proviso of a limited number of identified relationships and their weak statistical strength. However, the described relationships confirmed the links between diet and the health status of e-sports players, assuming that health status is determined by multiple factors and that diet is one of the predictive factors. The described nutritional and health relationships are justified by the nutritional value and health benefits of the food product groups defining the analyzed diet quality indicators, as well as by the functional properties of the nutrients sourced from these product groups. Thus, the positive relationship between the healthy diet index (pHDI-10) and blood vitamin D concentration can be explained by the nutritional value of sea fish and dairy products, which are important dietary sources of vitamin D. The positive relationship between the healthy diet index (pHDI-10) and HDL cholesterol concentration may be attributed to the consumption of sea fish as well as vegetables, fruit, whole-grain cereal products and fermented dairy products. These foods provide omega-3 polyunsaturated fatty acids, antioxidant vitamins, polyphenols, dietary fibre and probiotic compounds, which exert beneficial effects on lipid metabolism and contribute to improved blood lipid profiles due to their hypolipidemic properties (63).

The observed negative associations between the healthy diet index (pHDI-10), overall diet quality (DQI-24) and fasting uric acid and glucose levels may be explained by higher intake of low- and medium-glycaemic foods rich in antioxidants and dietary fibre, particularly vegetables, fruit and whole-grain products. Importantly, overweight and obesity constitute major risk factors for hyperuricemia, largely due to insulin resistance and impaired renal uric acid excretion. Hyperuricemia is further associated with excessive consumption of foods rich in purines and simple sugars, such as red meat, processed meat, offal, alcoholic beverages, confectionery and sugar-sweetened beverages (64). Similarly, hyperglycaemia is promoted by diets characterized by a high glycaemic index and load combined with low dietary fibre intake (65).

Due to the limited number of studies on the relationship between diet and health in e-sports players, discussion of the results may be limited. Previous studies in this area in e-sports players have confirmed positive associations between the frequency of consumption of products of low health quality, including those rich in simple sugars (sweet cereals) and saturated fatty acids and cholesterol (red meat and meat products), and zonulin levels in feces (36). Studies in this area have generally shown the positive effects of a diet with high nutrient density (rich in polyphenols, omega-3 PUFAs, dietary fiber and probiotics) and a negative effect of a low-nutrient-density diet (rich in processed foods, saturated fatty acids and simple sugars) on the gut microbiota (66). In this context, it is worth mentioning the Mediterranean diet, which has a positive effect on the state of the gut microbiota (25). However, it should be added that the results of some studies have not confirmed the relationship between diet quality and the state of the gut microbiota in athletes (67), which corresponds to the results of our own studies. The positive relationship between the Mediterranean diet and the health of athletes, including lower body fat, has been demonstrated in new meta-analyses (68). Other studies have confirmed the relationship between dietary patterns, gut microbiota and athletic performance. It has been found that following specific dietary patterns (e.g., the Mediterranean diet) can improve blood vessel function, reduce the risk of developing chronic diseases, and support regeneration and weight control, thus indirectly promoting improved athletic performance (41). Korean studies have also confirmed the importance of the quality of a plant-based diet for blood lipid profiles (69), and Dutch studies have shown that a healthy plant-based diet significantly reduces the risk of metabolic syndrome, diabetes and cardiovascular disease in adults (70). The cited results of studies by other authors, indicating the positive impact of rational dietary models on the health of various population groups, although not directly, correspond with the results of our own research, suggesting positive associations between a higher quality diet and health in terms of several analyzed indicators, particularly those related to blood lipid profile and blood glucose and UA levels.

In summary, the presented research may contribute to further studies on the health determinants of e-sports players and, given the limited availability of work in this area, also serve as a point of reference. In the context of the established relationships between diet quality and certain health indicators, it seems reasonable to monitor the diet, nutritional status, and hematological, metabolic, and biochemical health indicators of e-sports players. These factors are interrelated and are crucial for the health and performance of e-sports players. A diet of high health quality seems to be associated with better health, which determines sporting success and a better quality of life.

### 4.4 Limitations

The limitations of the study are primarily related to the self-report nature of the dietary quality assessment tool, the lack of a control group, and the inclusion of only males, which prevents the generalization of the results and their application to a wider population. The limitations of this study are also related to the nature of the adopted dietary quality indicators, which do not take into account energy requirements and energy density of the diet, which may distort their interpretation in people with increased nutritional needs. Furthermore, these indicators may have insufficient discriminatory power to describe the relationship between diet and health status. Further research should take into account the limitations indicated, including a broader spectrum of factors determining health status, and therefore also other modifiable factors related to lifestyle, including psychological aspects such as coping with stress. Future studies should be planned to include a control group and women in order to enable the interpretation of results in relation to a wider population. In subsequent studies, alternative diet quality indicators, including those specifically developed for athletic populations (e.g., the Athlete Diet Index), may be applied.

## 5 Conclusion

The study group of Polish e-sports players showed a low level of dietary quality and varied levels of the analyzed health indicators, with normal hematological parameters and a prevalence of hyperglycemia, hyperuricemia, increased intestinal barrier permeability and excessive body weight.

Among the e-sports players surveyed, significant correlations were found between diet quality and certain health indicators, suggesting a positive impact of a high-quality diet on the health of e-sports players. It was shown that as the healthy diet index increased, vitamin D and HDL cholesterol levels increased, while uric acid and blood glucose levels decreased. Similarly, the overall diet quality index was negatively associated with uric acid and blood glucose concentrations. It should be added that the correlations found were weak, suggesting that caution should be exercised when drawing conclusions about the predictive role of diet quality for the health of e-sports players.

Health education and monitoring of diet quality and eating patterns, as well as somatic, hematological, metabolic and biochemical health indicators, seem to be justified for the early prevention of potential health risks among e-sports players.

## Data Availability

The raw data supporting the conclusions of this article will be made available by the authors, without undue reservation.
